# Henry Ward Beecher’s Sermons

**Published:** 1883-10

**Authors:** 


### HENRY WARD BEECHER'S SERMONS.

We copy the following from one of Mr. Beecher's printed sermons: "We are distinguished from the animal creation by the magnitude of the brain, by the complication and perfection of the nervous system; and the whole force of life—its emotional force, its thought force, its will force—resides in the nervous mass of the brain and in the nervous system of the body; and he who keeps that healthy is competent to all the functions of existence, and he who draws on that draws on his capital. A healthy nervous system is one in which every night a man makes up what has been expended in turning the wheel of industry through the day; is one in which food and sleep make up, every twenty-four hours, all that a man has wasted, and supplies some surplus.

"There are a variety of ways in which men draw off the vitality of their whole brain and nervous system. Excessive virtuous industry does it. It makes no difference whether a worm eats its way into a wooden cistern, or whether a hole is bored into it with a gimlet, or whether heat shrinks the stuff, its contents run out all the same; and no matter what it may be, anything that sets the nervous system aleak will drain it dry speedily, and leave a man a wreck. As soon as a man has wasted with riotous living, the substance that lies inside the brain and nervous system he is good for nothing. That is the vital part of every human being, it is the capital of a man's life; and when one has squandered that he is beggared and bankrupt beyond all description."

"The first thing we know, down goes one man paralyzed; down goes another man from softening of the brain, down goes another man with Bright's disease."

"PLYMOUTH PULPIT," a weekly publication of sermons preached by Henry Ward Beecher. Published by Fords, Howard & Hulbert, New York. Price $2.00 a year. HALL'S JOURNAL OF HEALTH, $1.00 a year. Both will be sent for $2.50.

---

DON'T GIVE ME MERCURY, said the bilious man. The patient quickly recovered, and out of gratitude handed the doctor an extra fee. Now, Doctor, said he, if I should be sick again and could not get you, what medicine shall I take? Ten grains of CALOMEL, said the Doctor.

BED-BUGS.—Take down the bedstead, and with a small paint brush work into the joints and cracks of the bedstead a mixture of half a teacupful of kerosene and a teaspoonful of paris green. This treatment is good for six to twelve months or more.

